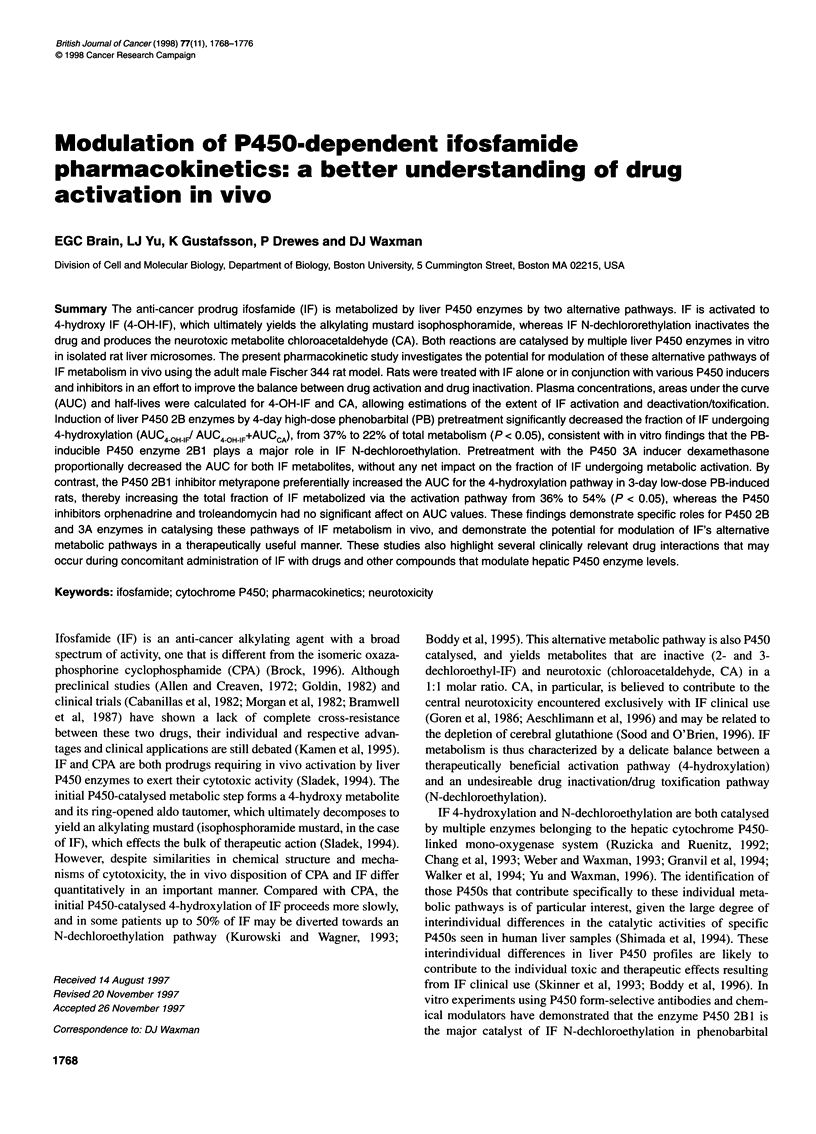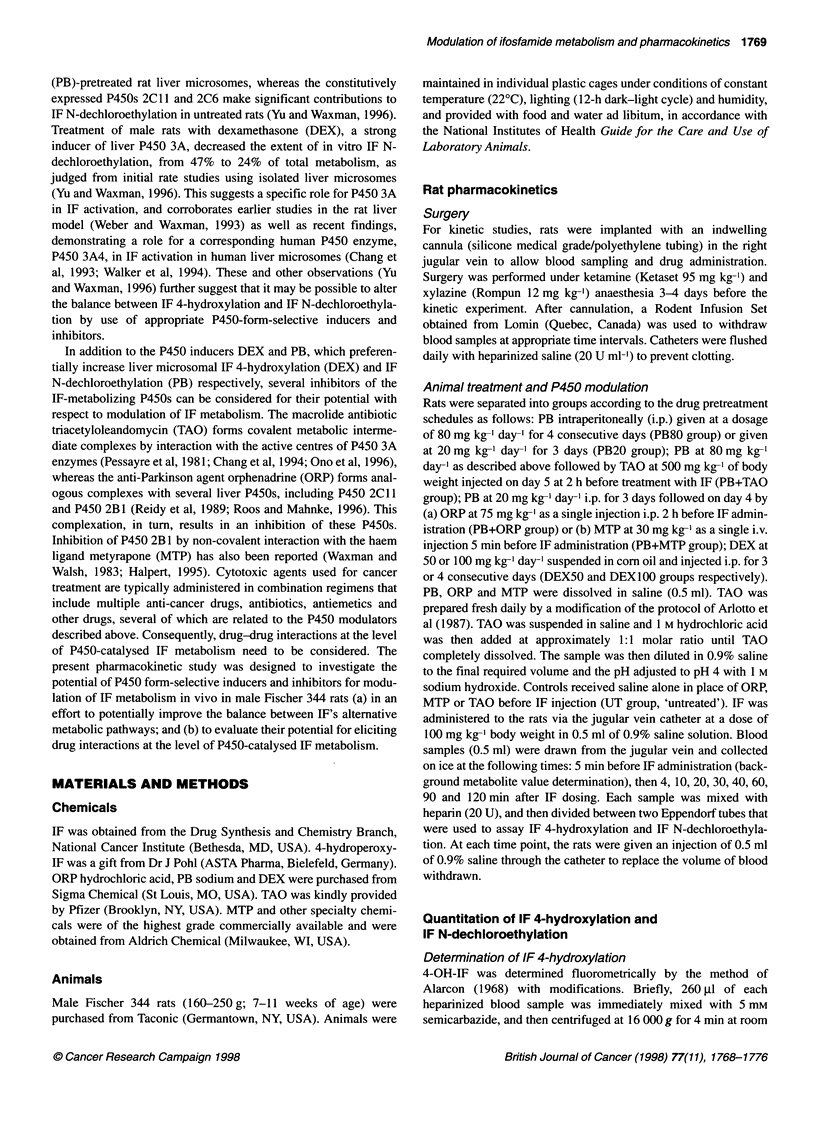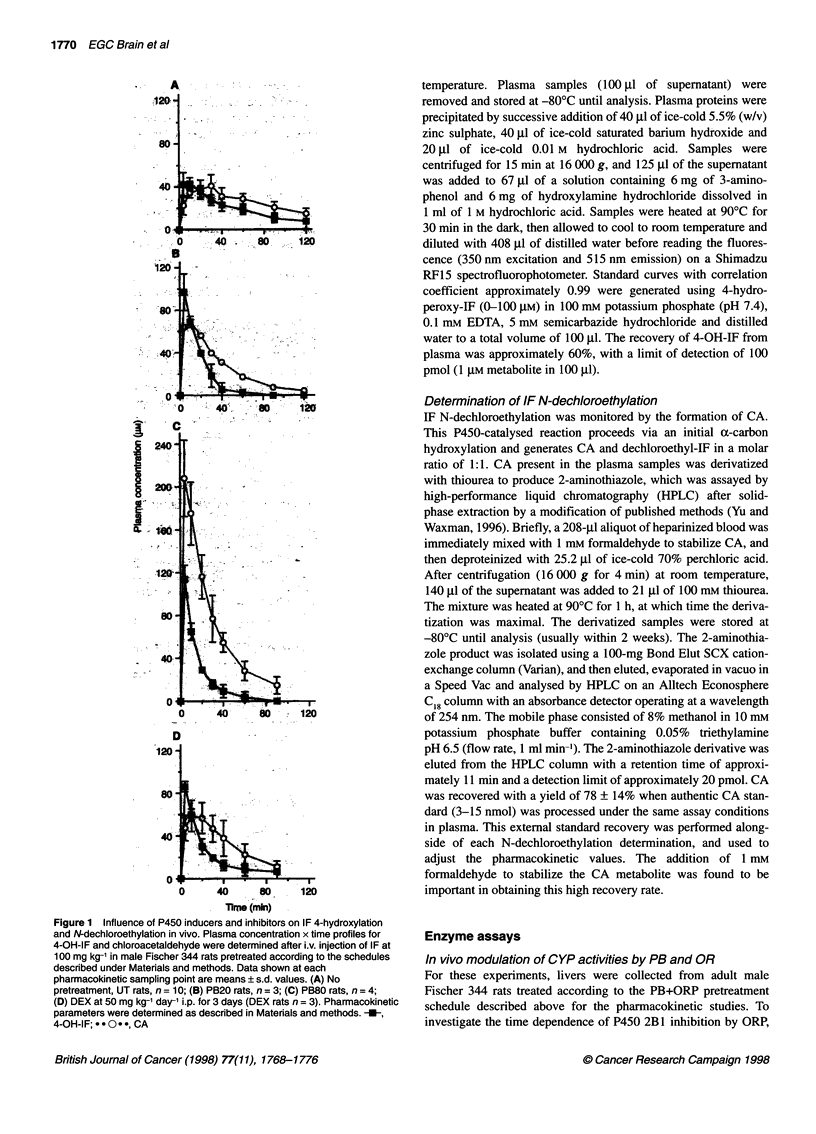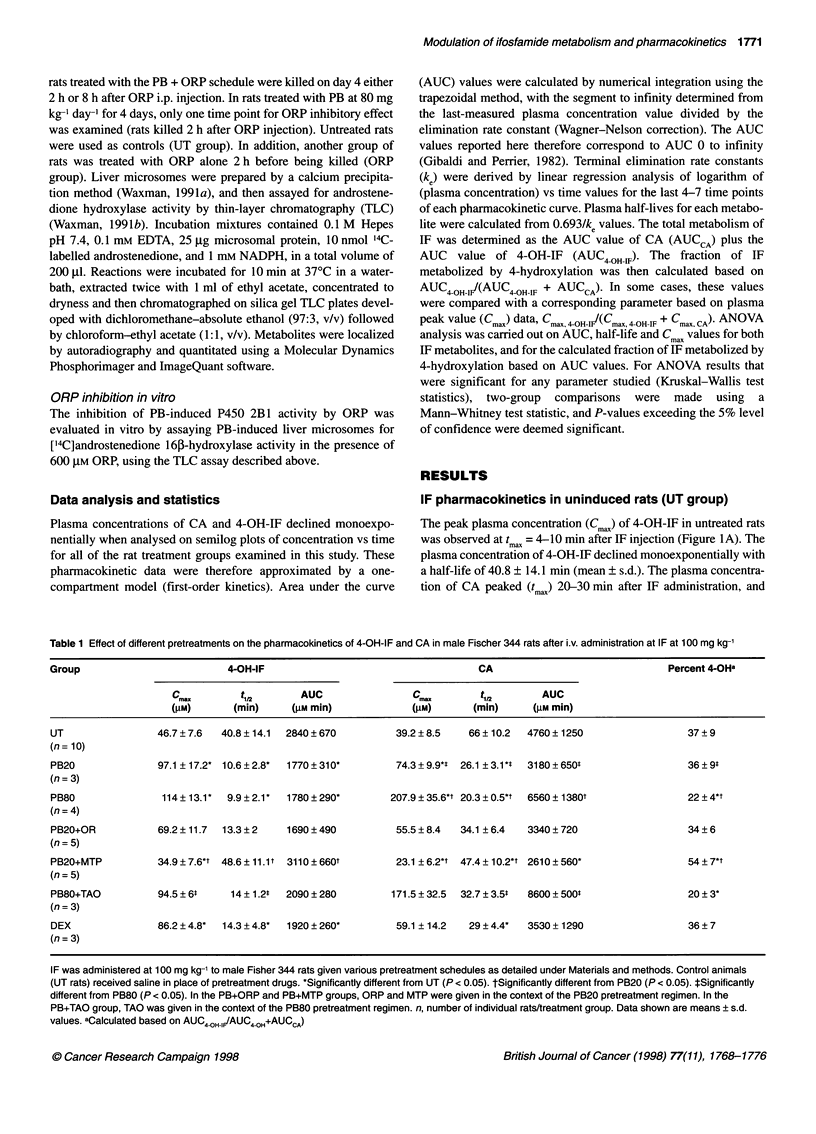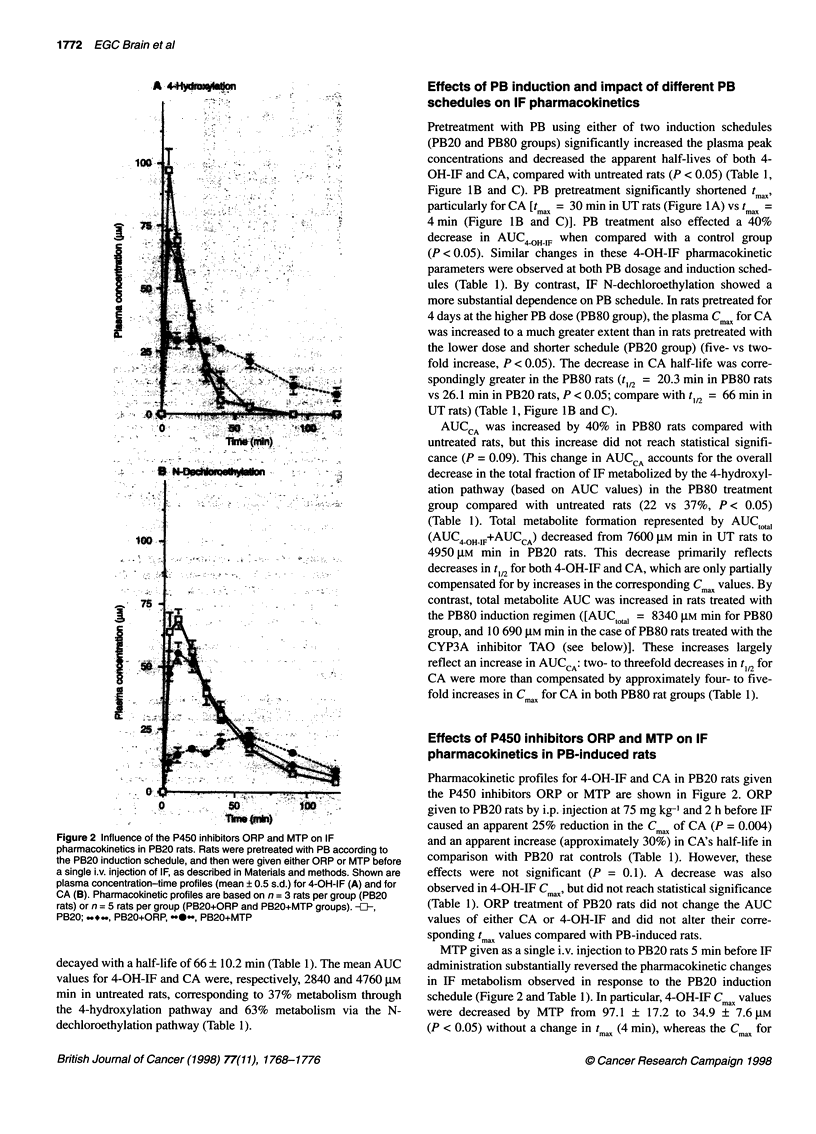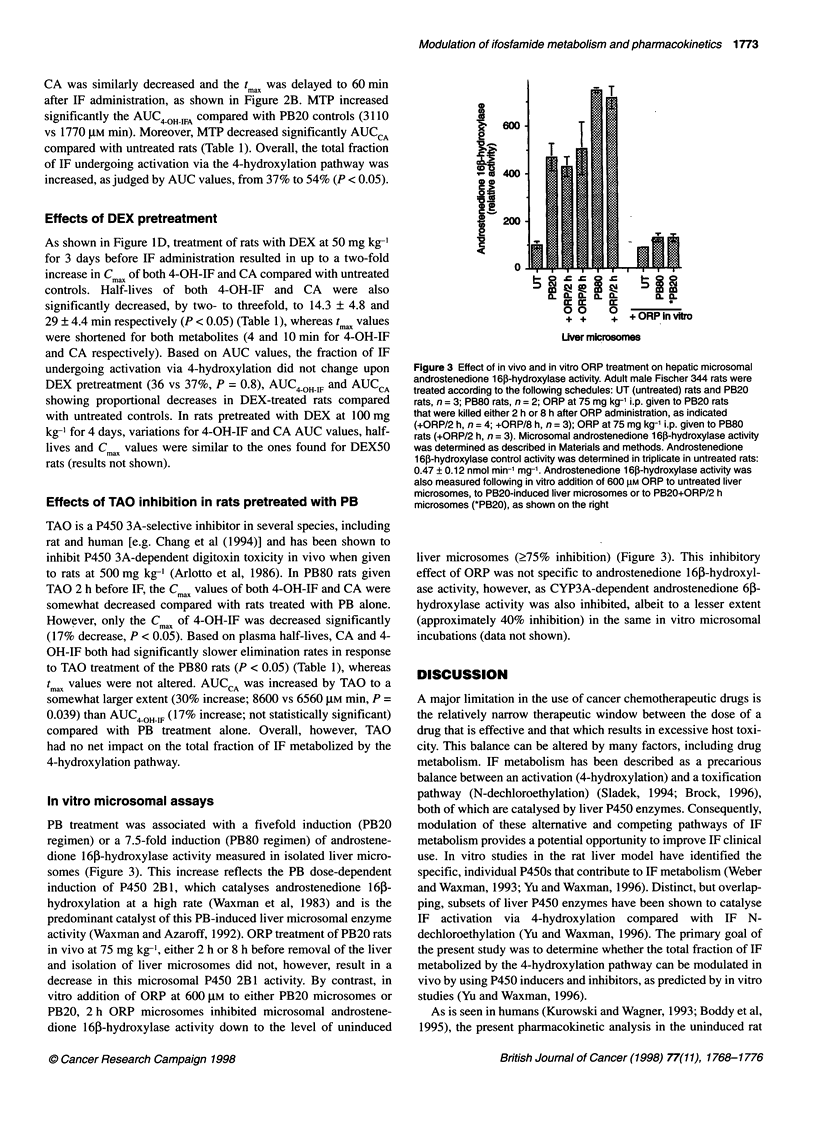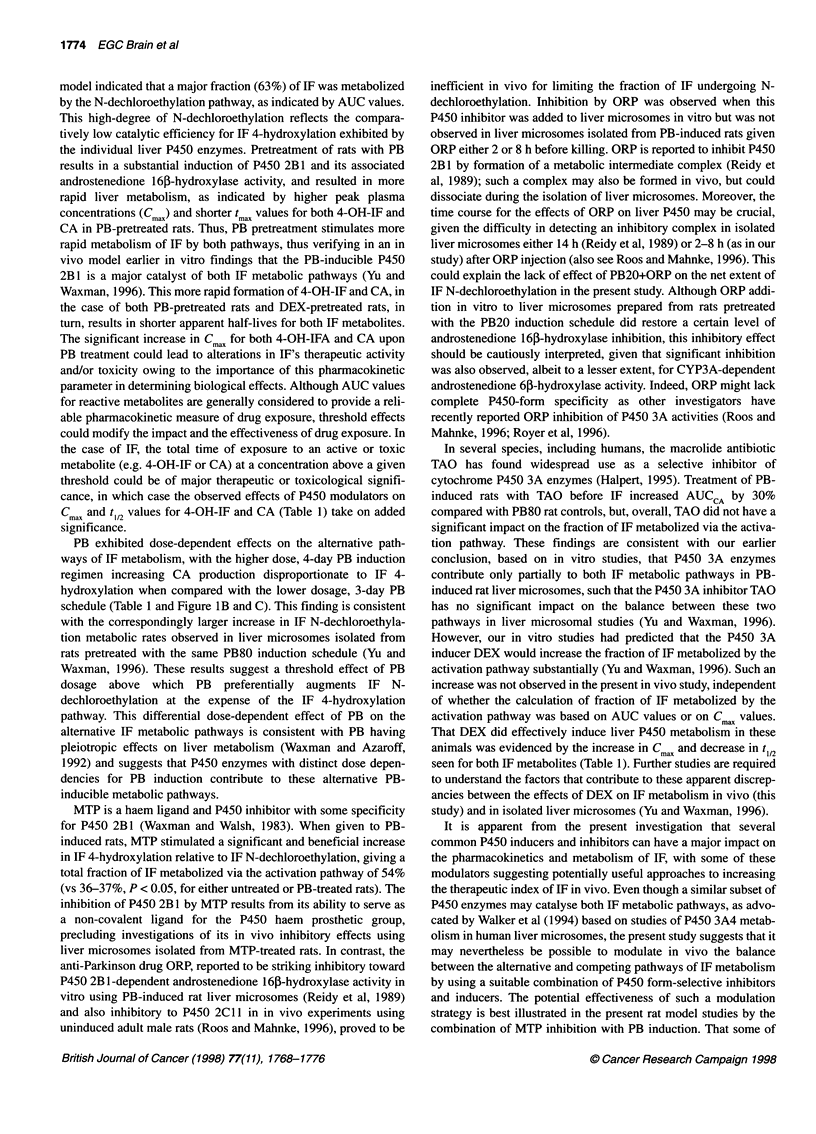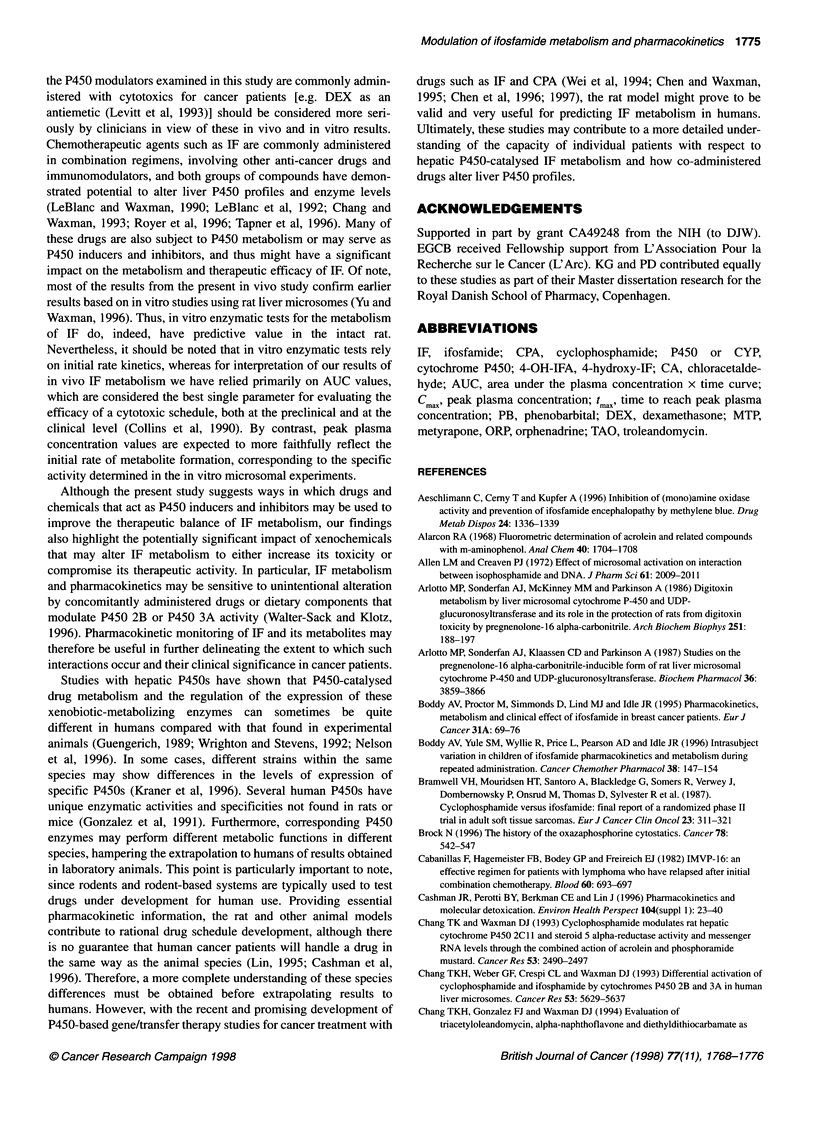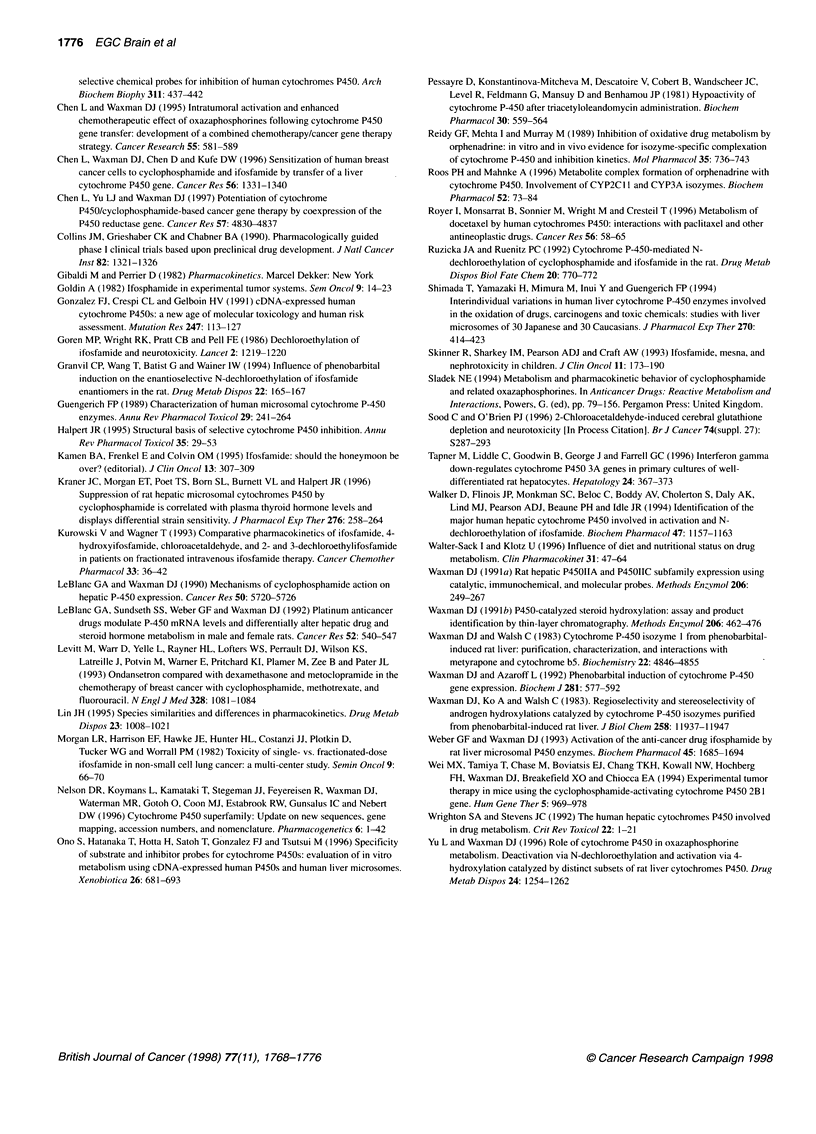# Modulation of P450-dependent ifosfamide pharmacokinetics: a better understanding of drug activation in vivo.

**DOI:** 10.1038/bjc.1998.295

**Published:** 1998-06

**Authors:** E. G. Brain, L. J. Yu, K. Gustafsson, P. Drewes, D. J. Waxman

**Affiliations:** Department of Biology, Boston University, MA 02215, USA.

## Abstract

The anti-cancer prodrug ifosfamide (IF) is metabolized by liver P450 enzymes by two alternative pathways. IF is activated to 4-hydroxy IF (4-OH-IF), which ultimately yields the alkylating mustard isophosphoramide, whereas IF N-dechlororethylation inactivates the drug and produces the neurotoxic metabolite chloroacetaldehyde (CA). Both reactions are catalysed by multiple liver P450 enzymes in vitro in isolated rat liver microsomes. The present pharmacokinetic study investigates the potential for modulation of these alternative pathways of IF metabolism in vivo using the adult male Fischer 344 rat model. Rats were treated with IF alone or in conjunction with various P450 inducers and inhibitors in an effort to improve the balance between drug activation and drug inactivation. Plasma concentrations, areas under the curve (AUC) and half-lives were calculated for 4-OH-IF and CA, allowing estimations of the extent of IF activation and deactivation/toxification. Induction of liver P450 2B enzymes by 4-day high-dose phenobarbital (PB) pretreatment significantly decreased the fraction of IF undergoing 4-hydroxylation (AUC(4-OH-IF)/AUC(4-OH-IF)+AUC(CA)), from 37% to 22% of total metabolism (P < 0.05), consistent with in vitro findings that the PB-inducible P450 enzyme 2B1 plays a major role in IF N-dechloroethylation. Pretreatment with the P450 3A inducer dexamethasone proportionally decreased the AUC for both IF metabolites, without any net impact on the fraction of IF undergoing metabolic activation. By contrast, the P450 2B1 inhibitor metyrapone preferentially increased the AUC for the 4-hydroxylation pathway in 3-day low-dose PB-induced rats, thereby increasing the total fraction of IF metabolized via the activation pathway from 36% to 54% (P < 0.05), whereas the P450 inhibitors orphenadrine and troleandomycin had no significant affect on AUC values. These findings demonstrate specific roles for P450 2B and 3A enzymes in catalysing these pathways of IF metabolism in vivo, and demonstrate the potential for modulation of IF's alternative metabolic pathways in a therapeutically useful manner. These studies also highlight several clinically relevant drug interactions that may occur during concomitant administration of IF with drugs and other compounds that modulate hepatic P450 enzyme levels.


British Journal of Cancer (1998) 77(11), 1768–1776
© 1998 Cancer Research Campaign

### Modulation of P450-dependent ifosfamide pharmacokinetics: a better understanding of drug activation in vivo

**EGC Brain, LJ Yu, K Gustafsson, P Drewes and DJ Waxman**

Division of Cell and Molecular Biology, Department of Biology, Boston University, 5 Cummington Street, Boston MA 02215, USA

**Summary** The anti-cancer prodrug ifosfamide (IF) is metabolized by liver P450 enzymes by two alternative pathways. IF is activated to 4-hydroxy IF (4-OH-IF), which ultimately yields the alkylating mustard isophosphoramide, whereas IF N-dechlororethylation inactivates the drug and produces the neurotoxic metabolite chloroacetaldehyde (CA). Both reactions are catalysed by multiple liver P450 enzymes in vitro in isolated rat liver microsomes. The present pharmacokinetic study investigates the potential for modulation of these alternative pathways of IF metabolism in vivo using the adult male Fischer 344 rat model. Rats were treated with IF alone or in conjunction with various P450 inducers and inhibitors in an effort to improve the balance between drug activation and drug inactivation. Plasma concentrations, areas under the curve (AUC) and half-lives were calculated for 4-OH-IF and CA, allowing estimations of the extent of IF activation and deactivation/toxification. Induction of liver P450 2B enzymes by 4-day high-dose phenobarbital (PB) pretreatment significantly decreased the fraction of IF undergoing 4-hydroxylation ($AUC_{4\text{-OH-IF}}/ AUC_{4\text{-OH-IF}}+AUC_{CA}$), from 37% to 22% of total metabolism ($P < 0.05$), consistent with in vitro findings that the PB-inducible P450 enzyme 2B1 plays a major role in IF N-dechloroethylation. Pretreatment with the P450 3A inducer dexamethasone proportionally decreased the AUC for both IF metabolites, without any net impact on the fraction of IF undergoing metabolic activation. By contrast, the P450 2B1 inhibitor metyrapone preferentially increased the AUC for the 4-hydroxylation pathway in 3-day low-dose PB-induced rats, thereby increasing the total fraction of IF metabolized via the activation pathway from 36% to 54% ($P < 0.05$), whereas the P450 inhibitors orphenadrine and troleandomycin had no significant affect on AUC values. These findings demonstrate specific roles for P450 2B and 3A enzymes in catalysing these pathways of IF metabolism in vivo, and demonstrate the potential for modulation of IF's alternative metabolic pathways in a therapeutically useful manner. These studies also highlight several clinically relevant drug interactions that may occur during concomitant administration of IF with drugs and other compounds that modulate hepatic P450 enzyme levels.

**Keywords:** ifosfamide; cytochrome P450; pharmacokinetics; neurotoxicity

Ifosfamide (IF) is an anti-cancer alkylating agent with a broad spectrum of activity, one that is different from the isomeric oxazaphosphorine cyclophosphamide (CPA) (Brock, 1996). Although preclinical studies (Allen and Creaven, 1972; Goldin, 1982) and clinical trials (Cabanillas et al, 1982; Morgan et al, 1982; Bramwell et al, 1987) have shown a lack of complete cross-resistance between these two drugs, their individual and respective advantages and clinical applications are still debated (Kamen et al, 1995). IF and CPA are both prodrugs requiring in vivo activation by liver P450 enzymes to exert their cytotoxic activity (Sladek, 1994). The initial P450-catalysed metabolic step forms a 4-hydroxy metabolite and its ring-opened aldo tautomer, which ultimately decomposes to yield an alkylating mustard (isophosphoramide mustard, in the case of IF), which effects the bulk of therapeutic action (Sladek, 1994). However, despite similarities in chemical structure and mechanisms of cytotoxicity, the in vivo disposition of CPA and IF differ quantitatively in an important manner. Compared with CPA, the initial P450-catalysed 4-hydroxylation of IF proceeds more slowly, and in some patients up to 50% of IF may be diverted towards an N-dechloroethylation pathway (Kurowski and Wagner, 1993; Boddy et al, 1995). This alternative metabolic pathway is also P450 catalysed, and yields metabolites that are inactive (2- and 3-dechloroethyl-IF) and neurotoxic (chloroacetaldehyde, CA) in a 1:1 molar ratio. CA, in particular, is believed to contribute to the central neurotoxicity encountered exclusively with IF clinical use (Goren et al, 1986; Aeschlimann et al, 1996) and may be related to the depletion of cerebral glutathione (Sood and O'Brien, 1996). IF metabolism is thus characterized by a delicate balance between a therapeutically beneficial activation pathway (4-hydroxylation) and an undesireable drug inactivation/drug toxification pathway (N-dechloroethylation).

IF 4-hydroxylation and N-dechloroethylation are both catalysed by multiple enzymes belonging to the hepatic cytochrome P450-linked mono-oxygenase system (Ruzicka and Ruenitz, 1992; Chang et al, 1993; Weber and Waxman, 1993; Granvil et al, 1994; Walker et al, 1994; Yu and Waxman, 1996). The identification of those P450s that contribute specifically to these individual metabolic pathways is of particular interest, given the large degree of interindividual differences in the catalytic activities of specific P450s seen in human liver samples (Shimada et al, 1994). These interindividual differences in liver P450 profiles are likely to contribute to the individual toxic and therapeutic effects resulting from IF clinical use (Skinner et al, 1993; Boddy et al, 1996). In vitro experiments using P450 form-selective antibodies and chemical modulators have demonstrated that the enzyme P450 2B1 is the major catalyst of IF N-dechloroethylation in phenobarbital

*Received 14 August 1997*
*Revised 20 November 1997*
*Accepted 26 November 1997*

*Correspondence to: DJ Waxman*

(PB)-pretreated rat liver microsomes, whereas the constitutively expressed P450s 2C11 and 2C6 make significant contributions to IF N-dechloroethylation in untreated rats (Yu and Waxman, 1996). Treatment of male rats with dexamethasone (DEX), a strong inducer of liver P450 3A, decreased the extent of in vitro IF N-dechloroethylation, from 47% to 24% of total metabolism, as judged from initial rate studies using isolated liver microsomes (Yu and Waxman, 1996). This suggests a specific role for P450 3A in IF activation, and corroborates earlier studies in the rat liver model (Weber and Waxman, 1993) as well as recent findings, demonstrating a role for a corresponding human P450 enzyme, P450 3A4, in IF activation in human liver microsomes (Chang et al, 1993; Walker et al, 1994). These and other observations (Yu and Waxman, 1996) further suggest that it may be possible to alter the balance between IF 4-hydroxylation and IF N-dechloroethylation by use of appropriate P450-form-selective inducers and inhibitors.

In addition to the P450 inducers DEX and PB, which preferentially increase liver microsomal IF 4-hydroxylation (DEX) and IF N-dechloroethylation (PB) respectively, several inhibitors of the IF-metabolizing P450s can be considered for their potential with respect to modulation of IF metabolism. The macrolide antibiotic triacetyloleandomycin (TAO) forms covalent metabolic intermediate complexes by interaction with the active centres of P450 3A enzymes (Pessayre et al, 1981; Chang et al, 1994; Ono et al, 1996), whereas the anti-Parkinson agent orphenadrine (ORP) forms analogous complexes with several liver P450s, including P450 2C11 and P450 2B1 (Reidy et al, 1989; Roos and Mahnke, 1996). This complexation, in turn, results in an inhibition of these P450s. Inhibition of P450 2B1 by non-covalent interaction with the haem ligand metyrapone (MTP) has also been reported (Waxman and Walsh, 1983; Halpert, 1995). Cytotoxic agents used for cancer treatment are typically administered in combination regimens that include multiple anti-cancer drugs, antibiotics, antiemetics and other drugs, several of which are related to the P450 modulators described above. Consequently, drug–drug interactions at the level of P450-catalysed IF metabolism need to be considered. The present pharmacokinetic study was designed to investigate the potential of P450 form-selective inducers and inhibitors for modulation of IF metabolism in vivo in male Fischer 344 rats (a) in an effort to potentially improve the balance between IF's alternative metabolic pathways; and (b) to evaluate their potential for eliciting drug interactions at the level of P450-catalysed IF metabolism.

### MATERIALS AND METHODS

### Chemicals

IF was obtained from the Drug Synthesis and Chemistry Branch, National Cancer Institute (Bethesda, MD, USA). 4-hydroperoxy-IF was a gift from Dr J Pohl (ASTA Pharma, Bielefeld, Germany). ORP hydrochloric acid, PB sodium and DEX were purchased from Sigma Chemical (St Louis, MO, USA). TAO was kindly provided by Pfizer (Brooklyn, NY, USA). MTP and other specialty chemicals were of the highest grade commercially available and were obtained from Aldrich Chemical (Milwaukee, WI, USA).

### Animals

Male Fischer 344 rats (160–250 g; 7–11 weeks of age) were purchased from Taconic (Germantown, NY, USA). Animals were maintained in individual plastic cages under conditions of constant temperature (22°C), lighting (12-h dark–light cycle) and humidity, and provided with food and water ad libitum, in accordance with the National Institutes of Health *Guide for the Care and Use of Laboratory Animals*.

### Rat pharmacokinetics

#### *Surgery*

For kinetic studies, rats were implanted with an indwelling cannula (silicone medical grade/polyethylene tubing) in the right jugular vein to allow blood sampling and drug administration. Surgery was performed under ketamine (Ketaset 95 mg $kg^{-1}$) and xylazine (Rompun 12 mg $kg^{-1}$) anaesthesia 3–4 days before the kinetic experiment. After cannulation, a Rodent Infusion Set obtained from Lomin (Quebec, Canada) was used to withdraw blood samples at appropriate time intervals. Catheters were flushed daily with heparinized saline (20 U $ml^{-1}$) to prevent clotting.

#### *Animal treatment and P450 modulation*

Rats were separated into groups according to the drug pretreatment schedules as follows: PB intraperitoneally (i.p.) given at a dosage of 80 mg $kg^{-1}$ $day^{-1}$ for 4 consecutive days (PB80 group) or given at 20 mg $kg^{-1}$ $day^{-1}$ for 3 days (PB20 group); PB at 80 mg $kg^{-1}$ $day^{-1}$ as described above followed by TAO at 500 mg $kg^{-1}$ of body weight injected on day 5 at 2 h before treatment with IF (PB+TAO group); PB at 20 mg $kg^{-1}$ $day^{-1}$ i.p. for 3 days followed on day 4 by (a) ORP at 75 mg $kg^{-1}$ as a single injection i.p. 2 h before IF administration (PB+ORP group) or (b) MTP at 30 mg $kg^{-1}$ as a single i.v. injection 5 min before IF administration (PB+MTP group); DEX at 50 or 100 mg $kg^{-1}$ $day^{-1}$ suspended in corn oil and injected i.p. for 3 or 4 consecutive days (DEX50 and DEX100 groups respectively). PB, ORP and MTP were dissolved in saline (0.5 ml). TAO was prepared fresh daily by a modification of the protocol of Arlotto et al (1987). TAO was suspended in saline and 1 M hydrochloric acid was then added at approximately 1:1 molar ratio until TAO completely dissolved. The sample was then diluted in 0.9% saline to the final required volume and the pH adjusted to pH 4 with 1 M sodium hydroxide. Controls received saline alone in place of ORP, MTP or TAO before IF injection (UT group, 'untreated'). IF was administered to the rats via the jugular vein catheter at a dose of 100 mg $kg^{-1}$ body weight in 0.5 ml of 0.9% saline solution. Blood samples (0.5 ml) were drawn from the jugular vein and collected on ice at the following times: 5 min before IF administration (background metabolite value determination), then 4, 10, 20, 30, 40, 60, 90 and 120 min after IF dosing. Each sample was mixed with heparin (20 U), and then divided between two Eppendorf tubes that were used to assay IF 4-hydroxylation and IF N-dechloroethylation. At each time point, the rats were given an injection of 0.5 ml of 0.9% saline through the catheter to replace the volume of blood withdrawn.

### Quantitation of IF 4-hydroxylation and IF N-dechloroethylation

#### *Determination of IF 4-hydroxylation*

4-OH-IF was determined fluorometrically by the method of Alarcon (1968) with modifications. Briefly, 260 µl of each heparinized blood sample was immediately mixed with 5 mM semicarbazide, and then centrifuged at 16 000 ***g*** for 4 min at room

© Cancer Research Campaign 1998

**Figure 1** Influence of P450 inducers and inhibitors on IF 4-hydroxylation and *N*-dechloroethylation in vivo. Plasma concentration × time profiles for 4-OH-IF and chloroacetaldehyde were determined after i.v. injection of IF at 100 mg kg$^{-1}$ in male Fischer 344 rats pretreated according to the schedules described under Materials and methods. Data shown at each pharmacokinetic sampling point are means ± s.d. values. (**A**) No pretreatment, UT rats, *n* = 10; (**B**) PB20 rats, *n* = 3; (**C**) PB80 rats, *n* = 4; (**D**) DEX at 50 mg kg$^{-1}$ day$^{-1}$ i.p. for 3 days (DEX rats *n* = 3). Pharmacokinetic parameters were determined as described in Materials and methods. –■–, 4-OH-IF; • • ○ • •, CA

temperature. Plasma samples (100 μl of supernatant) were removed and stored at –80°C until analysis. Plasma proteins were precipitated by successive addition of 40 μl of ice-cold 5.5% (w/v) zinc sulphate, 40 μl of ice-cold saturated barium hydroxide and 20 μl of ice-cold 0.01 M hydrochloric acid. Samples were centrifuged for 15 min at 16 000 ***g***, and 125 μl of the supernatant was added to 67 μl of a solution containing 6 mg of 3-aminophenol and 6 mg of hydroxylamine hydrochloride dissolved in 1 ml of 1 M hydrochloric acid. Samples were heated at 90°C for 30 min in the dark, then allowed to cool to room temperature and diluted with 408 μl of distilled water before reading the fluorescence (350 nm excitation and 515 nm emission) on a Shimadzu RF15 spectrofluorophotometer. Standard curves with correlation coefficient approximately 0.99 were generated using 4-hydroperoxy-IF (0–100 μM) in 100 mM potassium phosphate (pH 7.4), 0.1 mM EDTA, 5 mM semicarbazide hydrochloride and distilled water to a total volume of 100 μl. The recovery of 4-OH-IF from plasma was approximately 60%, with a limit of detection of 100 pmol (1 μM metabolite in 100 μl).

### *Determination of IF N-dechloroethylation*

IF N-dechloroethylation was monitored by the formation of CA. This P450-catalysed reaction proceeds via an initial α-carbon hydroxylation and generates CA and dechloroethyl-IF in a molar ratio of 1:1. CA present in the plasma samples was derivatized with thiourea to produce 2-aminothiazole, which was assayed by high-performance liquid chromatography (HPLC) after solid-phase extraction by a modification of published methods (Yu and Waxman, 1996). Briefly, a 208-μl aliquot of heparinized blood was immediately mixed with 1 mM formaldehyde to stabilize CA, and then deproteinized with 25.2 μl of ice-cold 70% perchloric acid. After centrifugation (16 000 ***g*** for 4 min) at room temperature, 140 μl of the supernatant was added to 21 μl of 100 mM thiourea. The mixture was heated at 90°C for 1 h, at which time the derivatization was maximal. The derivatized samples were stored at –80°C until analysis (usually within 2 weeks). The 2-aminothiazole product was isolated using a 100-mg Bond Elut SCX cation-exchange column (Varian), and then eluted, evaporated in vacuo in a Speed Vac and analysed by HPLC on an Alltech Econosphere $C_{18}$ column with an absorbance detector operating at a wavelength of 254 nm. The mobile phase consisted of 8% methanol in 10 mM potassium phosphate buffer containing 0.05% triethylamine pH 6.5 (flow rate, 1 ml min$^{-1}$). The 2-aminothiazole derivative was eluted from the HPLC column with a retention time of approximately 11 min and a detection limit of approximately 20 pmol. CA was recovered with a yield of 78 ± 14% when authentic CA standard (3–15 nmol) was processed under the same assay conditions in plasma. This external standard recovery was performed alongside of each N-dechloroethylation determination, and used to adjust the pharmacokinetic values. The addition of 1 mM formaldehyde to stabilize the CA metabolite was found to be important in obtaining this high recovery rate.

### Enzyme assays

### *In vivo modulation of CYP activities by PB and OR*

For these experiments, livers were collected from adult male Fischer 344 rats treated according to the PB+ORP pretreatment schedule described above for the pharmacokinetic studies. To investigate the time dependence of P450 2B1 inhibition by ORP,

© Cancer Research Campaign 1998

rats treated with the PB + ORP schedule were killed on day 4 either 2 h or 8 h after ORP i.p. injection. In rats treated with PB at 80 mg $kg^{-1}$ $day^{-1}$ for 4 days, only one time point for ORP inhibitory effect was examined (rats killed 2 h after ORP injection). Untreated rats were used as controls (UT group). In addition, another group of rats was treated with ORP alone 2 h before being killed (ORP group). Liver microsomes were prepared by a calcium precipitation method (Waxman, 1991*a*), and then assayed for androstenedione hydroxylase activity by thin-layer chromatography (TLC) (Waxman, 1991*b*). Incubation mixtures contained 0.1 M Hepes pH 7.4, 0.1 mM EDTA, 25 µg microsomal protein, 10 nmol $^{14}C$-labelled androstenedione, and 1 mM NADPH, in a total volume of 200 µl. Reactions were incubated for 10 min at 37°C in a water-bath, extracted twice with 1 ml of ethyl acetate, concentrated to dryness and then chromatographed on silica gel TLC plates developed with dichloromethane–absolute ethanol (97:3, v/v) followed by chloroform–ethyl acetate (1:1, v/v). Metabolites were localized by autoradiography and quantitated using a Molecular Dynamics Phosphorimager and ImageQuant software.

### ORP inhibition in vitro

The inhibition of PB-induced P450 2B1 activity by ORP was evaluated in vitro by assaying PB-induced liver microsomes for [$^{14}C$]androstenedione 16β-hydroxylase activity in the presence of 600 µM ORP, using the TLC assay described above.

### Data analysis and statistics

Plasma concentrations of CA and 4-OH-IF declined monoexponentially when analysed on semilog plots of concentration vs time for all of the rat treatment groups examined in this study. These pharmacokinetic data were therefore approximated by a one-compartment model (first-order kinetics). Area under the curve (AUC) values were calculated by numerical integration using the trapezoidal method, with the segment to infinity determined from the last-measured plasma concentration value divided by the elimination rate constant (Wagner–Nelson correction). The AUC values reported here therefore correspond to AUC 0 to infinity (Gibaldi and Perrier, 1982). Terminal elimination rate constants ($k_e$) were derived by linear regression analysis of logarithm of (plasma concentration) vs time values for the last 4–7 time points of each pharmacokinetic curve. Plasma half-lives for each metabolite were calculated from $0.693/k_e$ values. The total metabolism of IF was determined as the AUC value of CA ($AUC_{CA}$) plus the AUC value of 4-OH-IF ($AUC_{4\text{-OH-IF}}$). The fraction of IF metabolized by 4-hydroxylation was then calculated based on $AUC_{4\text{-OH-IF}}/(AUC_{4\text{-OH-IF}} + AUC_{CA})$. In some cases, these values were compared with a corresponding parameter based on plasma peak value ($C_{max}$) data, $C_{max,\,4\text{-OH-IF}}/(C_{max,\,4\text{-OH-IF}} + C_{max,\,CA})$. ANOVA analysis was carried out on AUC, half-life and $C_{max}$ values for both IF metabolites, and for the calculated fraction of IF metabolized by 4-hydroxylation based on AUC values. For ANOVA results that were significant for any parameter studied (Kruskal–Wallis test statistics), two-group comparisons were made using a Mann–Whitney test statistic, and *P*-values exceeding the 5% level of confidence were deemed significant.

### RESULTS

### IF pharmacokinetics in uninduced rats (UT group)

The peak plasma concentration ($C_{max}$) of 4-OH-IF in untreated rats was observed at $t_{max}$ = 4–10 min after IF injection (Figure 1A). The plasma concentration of 4-OH-IF declined monoexponentially with a half-life of 40.8 ± 14.1 min (mean ± s.d.). The plasma concentration of CA peaked ($t_{max}$) 20–30 min after IF administration, and

**Table 1** Effect of different pretreatments on the pharmacokinetics of 4-OH-IF and CA in male Fischer 344 rats after i.v. administration at IF at 100 mg $kg^{-1}$

| Group | 4-OH-IF | | | CA | | | Percent 4-OH[a] |
|---|---|---|---|---|---|---|---|
| | $C_{max}$ (µM) | $t_{1/2}$ (min) | AUC (µM min) | $C_{max}$ (µM) | $t_{1/2}$ (min) | AUC (µM min) | |
| UT (*n* = 10) | 46.7 ± 7.6 | 40.8 ± 14.1 | 2840 ± 670 | 39.2 ± 8.5 | 66 ± 10.2 | 4760 ± 1250 | 37 ± 9 |
| PB20 (*n* = 3) | 97.1 ± 17.2* | 10.6 ± 2.8* | 1770 ± 310* | 74.3 ± 9.9*‡ | 26.1 ± 3.1*‡ | 3180 ± 650‡ | 36 ± 9‡ |
| PB80 (*n* = 4) | 114 ± 13.1* | 9.9 ± 2.1* | 1780 ± 290* | 207.9 ± 35.6*† | 20.3 ± 0.5*† | 6560 ± 1380† | 22 ± 4*† |
| PB20+OR (*n* = 5) | 69.2 ± 11.7 | 13.3 ± 2 | 1690 ± 490 | 55.5 ± 8.4 | 34.1 ± 6.4 | 3340 ± 720 | 34 ± 6 |
| PB20+MTP (*n* = 5) | 34.9 ± 7.6*† | 48.6 ± 11.1† | 3110 ± 660† | 23.1 ± 6.2*† | 47.4 ± 10.2*† | 2610 ± 560* | 54 ± 7*† |
| PB80+TAO (*n* = 3) | 94.5 ± 6‡ | 14 ± 1.2‡ | 2090 ± 280 | 171.5 ± 32.5 | 32.7 ± 3.5‡ | 8600 ± 500‡ | 20 ± 3* |
| DEX (*n* = 3) | 86.2 ± 4.8* | 14.3 ± 4.8* | 1920 ± 260* | 59.1 ± 14.2 | 29 ± 4.4* | 3530 ± 1290 | 36 ± 7 |

IF was administered at 100 mg $kg^{-1}$ to male Fisher 344 rats given various pretreatment schedules as detailed under Materials and methods. Control animals (UT rats) received saline in place of pretreatment drugs. *Significantly different from UT ($P$ < 0.05). †Significantly different from PB20 ($P$ < 0.05). ‡Significantly different from PB80 ($P$ < 0.05). In the PB+ORP and PB+MTP groups, ORP and MTP were given in the context of the PB20 pretreatment regimen. In the PB+TAO group, TAO was given in the context of the PB80 pretreatment regimen. *n*, number of individual rats/treatment group. Data shown are means ± s.d. values. [a]Calculated based on $AUC_{4\text{-OH-IF}}/AUC_{4\text{-OH}}+AUC_{CA})$

© Cancer Research Campaign 1998

**Figure 2** Influence of the P450 inhibitors ORP and MTP on IF pharmacokinetics in PB20 rats. Rats were pretreated with PB according to the PB20 induction schedule, and then were given either ORP or MTP before a single i.v. injection of IF, as described in Materials and methods. Shown are plasma concentration–time profiles (mean ± 0.5 s.d.) for 4-OH-IF (**A**) and for CA (**B**). Pharmacokinetic profiles are based on $n = 3$ rats per group (PB20 rats) or $n = 5$ rats per group (PB20+ORP and PB20+MTP groups). -□-, PB20; ••♦••, PB20+ORP, ••●••, PB20+MTP

decayed with a half-life of 66 ± 10.2 min (Table 1). The mean AUC values for 4-OH-IF and CA were, respectively, 2840 and 4760 μM min in untreated rats, corresponding to 37% metabolism through the 4-hydroxylation pathway and 63% metabolism via the N-dechloroethylation pathway (Table 1).

### Effects of PB induction and impact of different PB schedules on IF pharmacokinetics

Pretreatment with PB using either of two induction schedules (PB20 and PB80 groups) significantly increased the plasma peak concentrations and decreased the apparent half-lives of both 4-OH-IF and CA, compared with untreated rats ($P < 0.05$) (Table 1, Figure 1B and C). PB pretreatment significantly shortened $t_{max}$, particularly for CA [$t_{max}$ = 30 min in UT rats (Figure 1A) vs $t_{max}$ = 4 min (Figure 1B and C)]. PB treatment also effected a 40% decrease in $AUC_{4\text{-OH-IF}}$ when compared with a control group ($P < 0.05$). Similar changes in these 4-OH-IF pharmacokinetic parameters were observed at both PB dosage and induction schedules (Table 1). By contrast, IF N-dechloroethylation showed a more substantial dependence on PB schedule. In rats pretreated for 4 days at the higher PB dose (PB80 group), the plasma $C_{max}$ for CA was increased to a much greater extent than in rats pretreated with the lower dose and shorter schedule (PB20 group) (five- vs two-fold increase, $P < 0.05$). The decrease in CA half-life was correspondingly greater in the PB80 rats ($t_{1/2}$ = 20.3 min in PB80 rats vs 26.1 min in PB20 rats, $P < 0.05$; compare with $t_{1/2}$ = 66 min in UT rats) (Table 1, Figure 1B and C).

$AUC_{CA}$ was increased by 40% in PB80 rats compared with untreated rats, but this increase did not reach statistical significance ($P = 0.09$). This change in $AUC_{CA}$ accounts for the overall decrease in the total fraction of IF metabolized by the 4-hydroxylation pathway (based on AUC values) in the PB80 treatment group compared with untreated rats (22 vs 37%, $P < 0.05$) (Table 1). Total metabolite formation represented by $AUC_{total}$ ($AUC_{4\text{-OH-IF}}$+$AUC_{CA}$) decreased from 7600 μM min in UT rats to 4950 μM min in PB20 rats. This decrease primarily reflects decreases in $t_{1/2}$ for both 4-OH-IF and CA, which are only partially compensated for by increases in the corresponding $C_{max}$ values. By contrast, total metabolite AUC was increased in rats treated with the PB80 induction regimen ([$AUC_{total}$ = 8340 μM min for PB80 group, and 10 690 μM min in the case of PB80 rats treated with the CYP3A inhibitor TAO (see below)]. These increases largely reflect an increase in $AUC_{CA}$: two- to threefold decreases in $t_{1/2}$ for CA were more than compensated by approximately four- to five-fold increases in $C_{max}$ for CA in both PB80 rat groups (Table 1).

### Effects of P450 inhibitors ORP and MTP on IF pharmacokinetics in PB-induced rats

Pharmacokinetic profiles for 4-OH-IF and CA in PB20 rats given the P450 inhibitors ORP or MTP are shown in Figure 2. ORP given to PB20 rats by i.p. injection at 75 mg $kg^{-1}$ and 2 h before IF caused an apparent 25% reduction in the $C_{max}$ of CA ($P = 0.004$) and an apparent increase (approximately 30%) in CA's half-life in comparison with PB20 rat controls (Table 1). However, these effects were not significant ($P = 0.1$). A decrease was also observed in 4-OH-IF $C_{max}$, but did not reach statistical significance (Table 1). ORP treatment of PB20 rats did not change the AUC values of either CA or 4-OH-IF and did not alter their corresponding $t_{max}$ values compared with PB-induced rats.

MTP given as a single i.v. injection to PB20 rats 5 min before IF administration substantially reversed the pharmacokinetic changes in IF metabolism observed in response to the PB20 induction schedule (Figure 2 and Table 1). In particular, 4-OH-IF $C_{max}$ values were decreased by MTP from 97.1 ± 17.2 to 34.9 ± 7.6 μM ($P < 0.05$) without a change in $t_{max}$ (4 min), whereas the $C_{max}$ for

© Cancer Research Campaign 1998

CA was similarly decreased and the $t_{max}$ was delayed to 60 min after IF administration, as shown in Figure 2B. MTP increased significantly the $AUC_{4\text{-}OH\text{-}IFA}$ compared with PB20 controls (3110 vs 1770 µM min). Moreover, MTP decreased significantly $AUC_{CA}$ compared with untreated rats (Table 1). Overall, the total fraction of IF undergoing activation via the 4-hydroxylation pathway was increased, as judged by AUC values, from 37% to 54% ($P < 0.05$).

### Effects of DEX pretreatment

As shown in Figure 1D, treatment of rats with DEX at 50 mg $kg^{-1}$ for 3 days before IF administration resulted in up to a two-fold increase in $C_{max}$ of both 4-OH-IF and CA compared with untreated controls. Half-lives of both 4-OH-IF and CA were also significantly decreased, by two- to threefold, to 14.3 ± 4.8 and 29 ± 4.4 min respectively ($P < 0.05$) (Table 1), whereas $t_{max}$ values were shortened for both metabolites (4 and 10 min for 4-OH-IF and CA respectively). Based on AUC values, the fraction of IF undergoing activation via 4-hydroxylation did not change upon DEX pretreatment (36 vs 37%, $P = 0.8$), $AUC_{4\text{-}OH\text{-}IF}$ and $AUC_{CA}$ showing proportional decreases in DEX-treated rats compared with untreated controls. In rats pretreated with DEX at 100 mg $kg^{-1}$ for 4 days, variations for 4-OH-IF and CA AUC values, half-lives and $C_{max}$ values were similar to the ones found for DEX50 rats (results not shown).

### Effects of TAO inhibition in rats pretreated with PB

TAO is a P450 3A-selective inhibitor in several species, including rat and human [e.g. Chang et al (1994)] and has been shown to inhibit P450 3A-dependent digitoxin toxicity in vivo when given to rats at 500 mg $kg^{-1}$ (Arlotto et al, 1986). In PB80 rats given TAO 2 h before IF, the $C_{max}$ values of both 4-OH-IF and CA were somewhat decreased compared with rats treated with PB alone. However, only the $C_{max}$ of 4-OH-IF was decreased significantly (17% decrease, $P < 0.05$). Based on plasma half-lives, CA and 4-OH-IF both had significantly slower elimination rates in response to TAO treatment of the PB80 rats ($P < 0.05$) (Table 1), whereas $t_{max}$ values were not altered. $AUC_{CA}$ was increased by TAO to a somewhat larger extent (30% increase; 8600 vs 6560 µM min, $P = 0.039$) than $AUC_{4\text{-}OH\text{-}IF}$ (17% increase; not statistically significant) compared with PB treatment alone. Overall, however, TAO had no net impact on the total fraction of IF metabolized by the 4-hydroxylation pathway.

### In vitro microsomal assays

PB treatment was associated with a fivefold induction (PB20 regimen) or a 7.5-fold induction (PB80 regimen) of androstenedione 16β-hydroxylase activity measured in isolated liver microsomes (Figure 3). This increase reflects the PB dose-dependent induction of P450 2B1, which catalyses androstenedione 16β-hydroxylation at a high rate (Waxman et al, 1983) and is the predominant catalyst of this PB-induced liver microsomal enzyme activity (Waxman and Azaroff, 1992). ORP treatment of PB20 rats in vivo at 75 mg $kg^{-1}$, either 2 h or 8 h before removal of the liver and isolation of liver microsomes did not, however, result in a decrease in this microsomal P450 2B1 activity. By contrast, in vitro addition of ORP at 600 µM to either PB20 microsomes or PB20, 2 h ORP microsomes inhibited microsomal androstenedione 16β-hydroxylase activity down to the level of uninduced

**Figure 3** Effect of in vivo and in vitro ORP treatment on hepatic microsomal androstenedione 16β-hydroxylase activity. Adult male Fischer 344 rats were treated according to the following schedules: UT (untreated) rats and PB20 rats, $n = 3$; PB80 rats, $n = 2$; ORP at 75 mg $kg^{-1}$ i.p. given to PB20 rats that were killed either 2 h or 8 h after ORP administration, as indicated (+ORP/2 h, $n = 4$; +ORP/8 h, $n = 3$); ORP at 75 mg $kg^{-1}$ i.p. given to PB80 rats (+ORP/2 h, $n = 3$). Microsomal androstenedione 16β-hydroxylase activity was determined as described in Materials and methods. Androstenedione 16β-hydroxylase control activity was determined in triplicate in untreated rats: 0.47 ± 0.12 nmol $min^{-1}$ $mg^{-1}$. Androstenedione 16β-hydroxylase activity was also measured following in vitro addition of 600 µM ORP to untreated liver microsomes, to PB20-induced liver microsomes or to PB20+ORP/2 h microsomes (*PB20), as shown on the right liver microsomes (≥75% inhibition) (Figure 3). This inhibitory effect of ORP was not specific to androstenedione 16β-hydroxylase activity, however, as CYP3A-dependent androstenedione 6β-hydroxylase activity was also inhibited, albeit to a lesser extent (approximately 40% inhibition) in the same in vitro microsomal incubations (data not shown).

### DISCUSSION

A major limitation in the use of cancer chemotherapeutic drugs is the relatively narrow therapeutic window between the dose of a drug that is effective and that which results in excessive host toxicity. This balance can be altered by many factors, including drug metabolism. IF metabolism has been described as a precarious balance between an activation (4-hydroxylation) and a toxification pathway (N-dechloroethylation) (Sladek, 1994; Brock, 1996), both of which are catalysed by liver P450 enzymes. Consequently, modulation of these alternative and competing pathways of IF metabolism provides a potential opportunity to improve IF clinical use. In vitro studies in the rat liver model have identified the specific, individual P450s that contribute to IF metabolism (Weber and Waxman, 1993; Yu and Waxman, 1996). Distinct, but overlapping, subsets of liver P450 enzymes have been shown to catalyse IF activation via 4-hydroxylation compared with IF N-dechloroethylation (Yu and Waxman, 1996). The primary goal of the present study was to determine whether the total fraction of IF metabolized by the 4-hydroxylation pathway can be modulated in vivo by using P450 inducers and inhibitors, as predicted by in vitro studies (Yu and Waxman, 1996).

As is seen in humans (Kurowski and Wagner, 1993; Boddy et al, 1995), the present pharmacokinetic analysis in the uninduced rat

*© Cancer Research Campaign 1998*

model indicated that a major fraction (63%) of IF was metabolized by the N-dechloroethylation pathway, as indicated by AUC values. This high-degree of N-dechloroethylation reflects the comparatively low catalytic efficiency for IF 4-hydroxylation exhibited by the individual liver P450 enzymes. Pretreatment of rats with PB results in a substantial induction of P450 2B1 and its associated androstenedione 16β-hydroxylase activity, and resulted in more rapid liver metabolism, as indicated by higher peak plasma concentrations ($C_{max}$) and shorter $t_{max}$ values for both 4-OH-IF and CA in PB-pretreated rats. Thus, PB pretreatment stimulates more rapid metabolism of IF by both pathways, thus verifying in an in vivo model earlier in vitro findings that the PB-inducible P450 2B1 is a major catalyst of both IF metabolic pathways (Yu and Waxman, 1996). This more rapid formation of 4-OH-IF and CA, in the case of both PB-pretreated rats and DEX-pretreated rats, in turn, results in shorter apparent half-lives for both IF metabolites. The significant increase in $C_{max}$ for both 4-OH-IFA and CA upon PB treatment could lead to alterations in IF's therapeutic activity and/or toxicity owing to the importance of this pharmacokinetic parameter in determining biological effects. Although AUC values for reactive metabolites are generally considered to provide a reliable pharmacokinetic measure of drug exposure, threshold effects could modify the impact and the effectiveness of drug exposure. In the case of IF, the total time of exposure to an active or toxic metabolite (e.g. 4-OH-IF or CA) at a concentration above a given threshold could be of major therapeutic or toxicological significance, in which case the observed effects of P450 modulators on $C_{max}$ and $t_{1/2}$ values for 4-OH-IF and CA (Table 1) take on added significance.

PB exhibited dose-dependent effects on the alternative pathways of IF metabolism, with the higher dose, 4-day PB induction regimen increasing CA production disproportionate to IF 4-hydroxylation when compared with the lower dosage, 3-day PB schedule (Table 1 and Figure 1B and C). This finding is consistent with the correspondingly larger increase in IF N-dechloroethylation metabolic rates observed in liver microsomes isolated from rats pretreated with the same PB80 induction schedule (Yu and Waxman, 1996). These results suggest a threshold effect of PB dosage above which PB preferentially augments IF N-dechloroethylation at the expense of the IF 4-hydroxylation pathway. This differential dose-dependent effect of PB on the alternative IF metabolic pathways is consistent with PB having pleiotropic effects on liver metabolism (Waxman and Azaroff, 1992) and suggests that P450 enzymes with distinct dose dependencies for PB induction contribute to these alternative PB-inducible metabolic pathways.

MTP is a haem ligand and P450 inhibitor with some specificity for P450 2B1 (Waxman and Walsh, 1983). When given to PB-induced rats, MTP stimulated a significant and beneficial increase in IF 4-hydroxylation relative to IF N-dechloroethylation, giving a total fraction of IF metabolized via the activation pathway of 54% (vs 36–37%, $P < 0.05$, for either untreated or PB-treated rats). The inhibition of P450 2B1 by MTP results from its ability to serve as a non-covalent ligand for the P450 haem prosthetic group, precluding investigations of its in vivo inhibitory effects using liver microsomes isolated from MTP-treated rats. In contrast, the anti-Parkinson drug ORP, reported to be striking inhibitory toward P450 2B1-dependent androstenedione 16β-hydroxylase activity in vitro using PB-induced rat liver microsomes (Reidy et al, 1989) and also inhibitory to P450 2C11 in in vivo experiments using uninduced adult male rats (Roos and Mahnke, 1996), proved to be inefficient in vivo for limiting the fraction of IF undergoing N-dechloroethylation. Inhibition by ORP was observed when this P450 inhibitor was added to liver microsomes in vitro but was not observed in liver microsomes isolated from PB-induced rats given ORP either 2 or 8 h before killing. ORP is reported to inhibit P450 2B1 by formation of a metabolic intermediate complex (Reidy et al, 1989); such a complex may also be formed in vivo, but could dissociate during the isolation of liver microsomes. Moreover, the time course for the effects of ORP on liver P450 may be crucial, given the difficulty in detecting an inhibitory complex in isolated liver microsomes either 14 h (Reidy et al, 1989) or 2–8 h (as in our study) after ORP injection (also see Roos and Mahnke, 1996). This could explain the lack of effect of PB20+ORP on the net extent of IF N-dechloroethylation in the present study. Although ORP addition in vitro to liver microsomes prepared from rats pretreated with the PB20 induction schedule did restore a certain level of androstenedione 16β-hydroxylase inhibition, this inhibitory effect should be cautiously interpreted, given that significant inhibition was also observed, albeit to a lesser extent, for CYP3A-dependent androstenedione 6β-hydroxylase activity. Indeed, ORP might lack complete P450-form specificity as other investigators have recently reported ORP inhibition of P450 3A activities (Roos and Mahnke, 1996; Royer et al, 1996).

In several species, including humans, the macrolide antibiotic TAO has found widespread use as a selective inhibitor of cytochrome P450 3A enzymes (Halpert, 1995). Treatment of PB-induced rats with TAO before IF increased $AUC_{CA}$ by 30% compared with PB80 rat controls, but, overall, TAO did not have a significant impact on the fraction of IF metabolized via the activation pathway. These findings are consistent with our earlier conclusion, based on in vitro studies, that P450 3A enzymes contribute only partially to both IF metabolic pathways in PB-induced rat liver microsomes, such that the P450 3A inhibitor TAO has no significant impact on the balance between these two pathways in liver microsomal studies (Yu and Waxman, 1996). However, our in vitro studies had predicted that the P450 3A inducer DEX would increase the fraction of IF metabolized by the activation pathway substantially (Yu and Waxman, 1996). Such an increase was not observed in the present in vivo study, independent of whether the calculation of fraction of IF metabolized by the activation pathway was based on AUC values or on $C_{max}$ values. That DEX did effectively induce liver P450 metabolism in these animals was evidenced by the increase in $C_{max}$ and decrease in $t_{1/2}$ seen for both IF metabolites (Table 1). Further studies are required to understand the factors that contribute to these apparent discrepancies between the effects of DEX on IF metabolism in vivo (this study) and in isolated liver microsomes (Yu and Waxman, 1996).

It is apparent from the present investigation that several common P450 inducers and inhibitors can have a major impact on the pharmacokinetics and metabolism of IF, with some of these modulators suggesting potentially useful approaches to increasing the therapeutic index of IF in vivo. Even though a similar subset of P450 enzymes may catalyse both IF metabolic pathways, as advocated by Walker et al (1994) based on studies of P450 3A4 metabolism in human liver microsomes, the present study suggests that it may nevertheless be possible to modulate in vivo the balance between the alternative and competing pathways of IF metabolism by using a suitable combination of P450 form-selective inhibitors and inducers. The potential effectiveness of such a modulation strategy is best illustrated in the present rat model studies by the combination of MTP inhibition with PB induction. That some of

 © Cancer Research Campaign 1998

the P450 modulators examined in this study are commonly administered with cytotoxics for cancer patients [e.g. DEX as an antiemetic (Levitt et al, 1993)] should be considered more seriously by clinicians in view of these in vivo and in vitro results. Chemotherapeutic agents such as IF are commonly administered in combination regimens, involving other anti-cancer drugs and immunomodulators, and both groups of compounds have demonstrated potential to alter liver P450 profiles and enzyme levels (LeBlanc and Waxman, 1990; LeBlanc et al, 1992; Chang and Waxman, 1993; Royer et al, 1996; Tapner et al, 1996). Many of these drugs are also subject to P450 metabolism or may serve as P450 inducers and inhibitors, and thus might have a significant impact on the metabolism and therapeutic efficacy of IF. Of note, most of the results from the present in vivo study confirm earlier results based on in vitro studies using rat liver microsomes (Yu and Waxman, 1996). Thus, in vitro enzymatic tests for the metabolism of IF do, indeed, have predictive value in the intact rat. Nevertheless, it should be noted that in vitro enzymatic tests rely on initial rate kinetics, whereas for interpretation of our results of in vivo IF metabolism we have relied primarily on AUC values, which are considered the best single parameter for evaluating the efficacy of a cytotoxic schedule, both at the preclinical and at the clinical level (Collins et al, 1990). By contrast, peak plasma concentration values are expected to more faithfully reflect the initial rate of metabolite formation, corresponding to the specific activity determined in the in vitro microsomal experiments.

Although the present study suggests ways in which drugs and chemicals that act as P450 inducers and inhibitors may be used to improve the therapeutic balance of IF metabolism, our findings also highlight the potentially significant impact of xenochemicals that may alter IF metabolism to either increase its toxicity or compromise its therapeutic activity. In particular, IF metabolism and pharmacokinetics may be sensitive to unintentional alteration by concomitantly administered drugs or dietary components that modulate P450 2B or P450 3A activity (Walter-Sack and Klotz, 1996). Pharmacokinetic monitoring of IF and its metabolites may therefore be useful in further delineating the extent to which such interactions occur and their clinical significance in cancer patients.

Studies with hepatic P450s have shown that P450-catalysed drug metabolism and the regulation of the expression of these xenobiotic-metabolizing enzymes can sometimes be quite different in humans compared with that found in experimental animals (Guengerich, 1989; Wrighton and Stevens, 1992; Nelson et al, 1996). In some cases, different strains within the same species may show differences in the levels of expression of specific P450s (Kraner et al, 1996). Several human P450s have unique enzymatic activities and specificities not found in rats or mice (Gonzalez et al, 1991). Furthermore, corresponding P450 enzymes may perform different metabolic functions in different species, hampering the extrapolation to humans of results obtained in laboratory animals. This point is particularly important to note, since rodents and rodent-based systems are typically used to test drugs under development for human use. Providing essential pharmacokinetic information, the rat and other animal models contribute to rational drug schedule development, although there is no guarantee that human cancer patients will handle a drug in the same way as the animal species (Lin, 1995; Cashman et al, 1996). Therefore, a more complete understanding of these species differences must be obtained before extrapolating results to humans. However, with the recent and promising development of P450-based gene/transfer therapy studies for cancer treatment with drugs such as IF and CPA (Wei et al, 1994; Chen and Waxman, 1995; Chen et al, 1996; 1997), the rat model might prove to be valid and very useful for predicting IF metabolism in humans. Ultimately, these studies may contribute to a more detailed understanding of the capacity of individual patients with respect to hepatic P450-catalysed IF metabolism and how co-administered drugs alter liver P450 profiles.

### ACKNOWLEDGEMENTS

Supported in part by grant CA49248 from the NIH (to DJW). EGCB received Fellowship support from L'Association Pour la Recherche sur le Cancer (L'Arc). KG and PD contributed equally to these studies as part of their Master dissertation research for the Royal Danish School of Pharmacy, Copenhagen.

### ABBREVIATIONS

IF, ifosfamide; CPA, cyclophosphamide; P450 or CYP, cytochrome P450; 4-OH-IFA, 4-hydroxy-IF; CA, chloracetaldehyde; AUC, area under the plasma concentration × time curve; $C_{max}$, peak plasma concentration; $t_{max}$, time to reach peak plasma concentration; PB, phenobarbital; DEX, dexamethasone; MTP, metyrapone, ORP, orphenadrine; TAO, troleandomycin.

### REFERENCES

Aeschlimann C, Cerny T and Kupfer A (1996) Inhibition of (mono)amine oxidase activity and prevention of ifosfamide encephalopathy by methylene blue. *Drug Metab Dispos* **24**: 1336–1339

Alarcon RA (1968) Fluorometric determination of acrolein and related compounds with m-aminophenol. *Anal Chem* **40**: 1704–1708

Allen LM and Creaven PJ (1972) Effect of microsomal activation on interaction between isophosphamide and DNA. *J Pharm Sci* **61**: 2009–2011

Arlotto MP, Sonderfan AJ, McKinney MM and Parkinson A (1986) Digitoxin metabolism by liver microsomal cytochrome P-450 and UDP-glucuronosyltransferase and its role in the protection of rats from digitoxin toxicity by pregnenolone-16 alpha-carbonitrile. *Arch Biochem Biophys* **251**: 188–197

Arlotto MP, Sonderfan AJ, Klaassen CD and Parkinson A (1987) Studies on the pregnenolone-16 alpha-carbonitrile-inducible form of rat liver microsomal cytochrome P-450 and UDP-glucuronosyltransferase. *Biochem Pharmacol* **36**: 3859–3866

Boddy AV, Proctor M, Simmonds D, Lind MJ and Idle JR (1995) Pharmacokinetics, metabolism and clinical effect of ifosfamide in breast cancer patients. *Eur J Cancer* **31A**: 69–76

Boddy AV, Yule SM, Wyllie R, Price L, Pearson AD and Idle JR (1996) Intrasubject variation in children of ifosfamide pharmacokinetics and metabolism during repeated administration. *Cancer Chemother Pharmacol* **38**: 147–154

Bramwell VH, Mouridsen HT, Santoro A, Blackledge G, Somers R, Verwey J, Dombernowsky P, Onsrud M, Thomas D, Sylvester R et al. (1987). Cyclophosphamide versus ifosfamide: final report of a randomized phase II trial in adult soft tissue sarcomas. *Eur J Cancer Clin Oncol* **23**: 311–321

Brock N (1996) The history of the oxazaphosphorine cytostatics. *Cancer* **78**: 542–547

Cabanillas F, Hagemeister FB, Bodey GP and Freireich EJ (1982) IMVP-16: an effective regimen for patients with lymphoma who have relapsed after initial combination chemotherapy. *Blood* **60**: 693–697

Cashman JR, Perotti BY, Berkman CE and Lin J (1996) Pharmacokinetics and molecular detoxication. *Environ Health Perspect* **104**(suppl 1): 23–40

Chang TK and Waxman DJ (1993) Cyclophosphamide modulates rat hepatic cytochrome P450 2C11 and steroid 5 alpha-reductase activity and messenger RNA levels through the combined action of acrolein and phosphoramide mustard. *Cancer Res* **53**: 2490–2497

Chang TKH, Weber GF, Crespi CL and Waxman DJ (1993) Differential activation of cyclophosphamide and ifosphamide by cytochromes P450 2B and 3A in human liver microsomes. *Cancer Res* **53**: 5629–5637

Chang TKH, Gonzalez FJ and Waxman DJ (1994) Evaluation of triacetyloleandomycin, alpha-naphthoflavone and diethyldithiocarbamate as

© Cancer Research Campaign 1998

selective chemical probes for inhibition of human cytochromes P450. *Arch Biochem Biophy* **311**: 437–442

Chen L and Waxman DJ (1995) Intratumoral activation and enhanced chemotherapeutic effect of oxazaphosphorines following cytochrome P450 gene transfer: development of a combined chemotherapy/cancer gene therapy strategy. *Cancer Research* **55**: 581–589

Chen L, Waxman DJ, Chen D and Kufe DW (1996) Sensitization of human breast cancer cells to cyclophosphamide and ifosfamide by transfer of a liver cytochrome P450 gene. *Cancer Res* **56**: 1331–1340

Chen L, Yu LJ and Waxman DJ (1997) Potentiation of cytochrome P450/cyclophosphamide-based cancer gene therapy by coexpression of the P450 reductase gene. *Cancer Res* **57**: 4830–4837

Collins JM, Grieshaber CK and Chabner BA (1990). Pharmacologically guided phase I clinical trials based upon preclinical drug development. *J Natl Cancer Inst* **82**: 1321–1326

Gibaldi M and Perrier D (1982) *Pharmacokinetics*. Marcel Dekker: New York

Goldin A (1982) Ifosphamide in experimental tumor systems. *Sem Oncol* **9**: 14–23

Gonzalez FJ, Crespi CL and Gelboin HV (1991) cDNA-expressed human cytochrome P450s: a new age of molecular toxicology and human risk assessment. *Mutation Res* **247**: 113–127

Goren MP, Wright RK, Pratt CB and Pell FE (1986) Dechloroethylation of ifosfamide and neurotoxicity. *Lancet* **2**: 1219–1220

Granvil CP, Wang T, Batist G and Wainer IW (1994) Influence of phenobarbital induction on the enantioselective N-dechloroethylation of ifosfamide enantiomers in the rat. *Drug Metab Dispos* **22**: 165–167

Guengerich FP (1989) Characterization of human microsomal cytochrome P-450 enzymes. *Annu Rev Pharmacol Toxicol* **29**: 241–264

Halpert JR (1995) Structural basis of selective cytochrome P450 inhibition. *Annu Rev Pharmacol Toxicol* **35**: 29–53

Kamen BA, Frenkel E and Colvin OM (1995) Ifosfamide: should the honeymoon be over? (editorial). *J Clin Oncol* **13**: 307–309

Kraner JC, Morgan ET, Poet TS, Born SL, Burnett VL and Halpert JR (1996) Suppression of rat hepatic microsomal cytochromes P450 by cyclophosphamide is correlated with plasma thyroid hormone levels and displays differential strain sensitivity. *J Pharmacol Exp Ther* **276**: 258–264

Kurowski V and Wagner T (1993) Comparative pharmacokinetics of ifosfamide, 4-hydroxyifosfamide, chloroacetaldehyde, and 2- and 3-dechloroethylifosfamide in patients on fractionated intravenous ifosfamide therapy. *Cancer Chemother Pharmacol* **33**: 36–42

LeBlanc GA and Waxman DJ (1990) Mechanisms of cyclophosphamide action on hepatic P-450 expression. *Cancer Res* **50**: 5720–5726

LeBlanc GA, Sundseth SS, Weber GF and Waxman DJ (1992) Platinum anticancer drugs modulate P-450 mRNA levels and differentially alter hepatic drug and steroid hormone metabolism in male and female rats. *Cancer Res* **52**: 540–547

Levitt M, Warr D, Yelle L, Rayner HL, Lofters WS, Perrault DJ, Wilson KS, Latreille J, Potvin M, Warner E, Pritchard KI, Plamer M, Zee B and Pater JL (1993) Ondansetron compared with dexamethasone and metoclopramide in the chemotherapy of breast cancer with cyclophosphamide, methotrexate, and fluorouracil. *N Engl J Med* **328**: 1081–1084

Lin JH (1995) Species similarities and differences in pharmacokinetics. *Drug Metab Dispos* **23**: 1008–1021

Morgan LR, Harrison EF, Hawke JE, Hunter HL, Costanzi JJ, Plotkin D, Tucker WG and Worrall PM (1982) Toxicity of single- vs. fractionated-dose ifosfamide in non-small cell lung cancer: a multi-center study. *Semin Oncol* **9**: 66–70

Nelson DR, Koymans L, Kamataki T, Stegeman JJ, Feyereisen R, Waxman DJ, Waterman MR, Gotoh O, Coon MJ, Estabrook RW, Gunsalus IC and Nebert DW (1996) Cytochrome P450 superfamily: Update on new sequences, gene mapping, accession numbers, and nomenclature. *Pharmacogenetics* **6**: 1–42

Ono S, Hatanaka T, Hotta H, Satoh T, Gonzalez FJ and Tsutsui M (1996) Specificity of substrate and inhibitor probes for cytochrome P450s: evaluation of in vitro metabolism using cDNA-expressed human P450s and human liver microsomes. *Xenobiotica* **26**: 681–693

Pessayre D, Konstantinova-Mitcheva M, Descatoire V, Cobert B, Wandscheer JC, Level R, Feldmann G, Mansuy D and Benhamou JP (1981) Hypoactivity of cytochrome P-450 after triacetyloleandomycin administration. *Biochem Pharmacol* **30**: 559–564

Reidy GF, Mehta I and Murray M (1989) Inhibition of oxidative drug metabolism by orphenadrine: in vitro and in vivo evidence for isozyme-specific complexation of cytochrome P-450 and inhibition kinetics. *Mol Pharmacol* **35**: 736–743

Roos PH and Mahnke A (1996) Metabolite complex formation of orphenadrine with cytochrome P450. Involvement of CYP2C11 and CYP3A isozymes. *Biochem Pharmacol* **52**: 73–84

Royer I, Monsarrat B, Sonnier M, Wright M and Cresteil T (1996) Metabolism of docetaxel by human cytochromes P450: interactions with paclitaxel and other antineoplastic drugs. *Cancer Res* **56**: 58–65

Ruzicka JA and Ruenitz PC (1992) Cytochrome P-450-mediated N-dechloroethylation of cyclophosphamide and ifosfamide in the rat. *Drug Metab Dispos Biol Fate Chem* **20**: 770–772

Shimada T, Yamazaki H, Mimura M, Inui Y and Guengerich FP (1994) Interindividual variations in human liver cytochrome P-450 enzymes involved in the oxidation of drugs, carcinogens and toxic chemicals: studies with liver microsomes of 30 Japanese and 30 Caucasians. *J Pharmacol Exp Ther* **270**: 414–423

Skinner R, Sharkey IM, Pearson ADJ and Craft AW (1993) Ifosfamide, mesna, and nephrotoxicity in children. *J Clin Oncol* **11**: 173–190

Sladek NE (1994) Metabolism and pharmacokinetic behavior of cyclophosphamide and related oxazaphosphorines. In *Anticancer Drugs: Reactive Metabolism and Interactions*, Powers, G. (ed), pp. 79–156. Pergamon Press: United Kingdom.

Sood C and O'Brien PJ (1996) 2-Chloroacetaldehyde-induced cerebral glutathione depletion and neurotoxicity [In Process Citation]. *Br J Cancer* **74**(suppl. 27): S287-293

Tapner M, Liddle C, Goodwin B, George J and Farrell GC (1996) Interferon gamma down-regulates cytochrome P450 3A genes in primary cultures of well-differentiated rat hepatocytes. *Hepatology* **24**: 367–373

Walker D, Flinois JP, Monkman SC, Beloc C, Boddy AV, Cholerton S, Daly AK, Lind MJ, Pearson ADJ, Beaune PH and Idle JR (1994) Identification of the major human hepatic cytochrome P450 involved in activation and N-dechloroethylation of ifosfamide. *Biochem Pharmacol* **47**: 1157–1163

Walter-Sack I and Klotz U (1996) Influence of diet and nutritional status on drug metabolism. *Clin Pharmacokinet* **31**: 47–64

Waxman DJ (1991*a*) Rat hepatic P450IIA and P450IIC subfamily expression using catalytic, immunochemical, and molecular probes. *Methods Enzymol* **206**: 249–267

Waxman DJ (1991*b*) P450-catalyzed steroid hydroxylation: assay and product identification by thin-layer chromatography. *Methods Enzymol* **206**: 462–476

Waxman DJ and Walsh C (1983) Cytochrome P-450 isozyme 1 from phenobarbital-induced rat liver: purification, characterization, and interactions with metyrapone and cytochrome b5. *Biochemistry* **22**: 4846–4855

Waxman DJ and Azaroff L (1992) Phenobarbital induction of cytochrome P-450 gene expression. *Biochem J* **281**: 577–592

Waxman DJ, Ko A and Walsh C (1983). Regioselectivity and stereoselectivity of androgen hydroxylations catalyzed by cytochrome P-450 isozymes purified from phenobarbital-induced rat liver. *J Biol Chem* **258**: 11937–11947

Weber GF and Waxman DJ (1993) Activation of the anti-cancer drug ifosphamide by rat liver microsomal P450 enzymes. *Biochem Pharmacol* **45**: 1685–1694

Wei MX, Tamiya T, Chase M, Boviatsis EJ, Chang TKH, Kowall NW, Hochberg FH, Waxman DJ, Breakefield XO and Chiocca EA (1994) Experimental tumor therapy in mice using the cyclophosphamide-activating cytochrome P450 2B1 gene. *Hum Gene Ther* **5**: 969–978

Wrighton SA and Stevens JC (1992) The human hepatic cytochromes P450 involved in drug metabolism. *Crit Rev Toxicol* **22**: 1–21

Yu L and Waxman DJ (1996) Role of cytochrome P450 in oxazaphosphorine metabolism. Deactivation via N-dechloroethylation and activation via 4-hydroxylation catalyzed by distinct subsets of rat liver cytochromes P450. *Drug Metab Dispos* **24**: 1254–1262

© Cancer Research Campaign 1998